# Identifying Genes Conferring Rust Resistance in Adzuki Bean (*Vigna angularis*) Through Genome-Wide Association Analysis Based on Genotyping-by-Sequencing (GBS)

**DOI:** 10.3390/plants15213449

**Published:** 2026-11-05

**Authors:** Xiaodan Xu, Zihan Zhang, Ningning Sun, Sian Peng, Tangyi Chen, Yanmin Qie, Lige Geng, Shihui Wang, Weina Sun, Yongxia Guo, Jidao Du

**Affiliations:** 1 College of Agronomy, Heilongjiang Bayi Agricultural University, Daqing 163319, China; xxdalice@163.com (X.X.); z18049253266@163.com (Z.Z.); 15046408530@163.com (N.S.); pengsian2026@163.com (S.P.);; 2 National Coarse Cereals Engineering Research Center, Daqing 163319, China; 3 Institute of Cereal and Oil Crops, Hebei Academy of Agricultural and Forestry Sciences/Hebei Key Laboratory of Crop Genetics and Breeding, Shijiazhuang 050035, China

**Keywords:** adzuki bean, genotyping-by-sequencing (GBS), genome-wide association analysis, rust, Kompetitive Allele-Specific PCR (KASP)

## Abstract

Adzuki bean (*Vigna angularis*) is an important pulse crop, and rust disease severely restricts its production. Identifying resistance genes is essential for accelerating the development of rust-resistant cultivars through marker-assisted breeding. In this study, genotyping-by-sequencing (GBS) was performed on 178 adzuki bean accessions, generating 124.87 Gb of sequencing data and yielding 12,153 high-quality single-nucleotide polymorphism (SNP) markers with an average density of 26.56 SNPs/Mb across the genome. Principal component analysis (PCA) and K-means clustering divided the 178 accessions into three distinct genetic clusters, with the first five principal components explaining 31.90% of the total genetic variation. Population structure analysis based on these SNPs revealed that the cross-entropy criterion reached its minimum at K = 7, partitioning the panel into seven genetic subpopulations (C1–C7). A neighbor-joining phylogenetic tree further resolved the accessions into five major groups. Rust resistance was assessed at the seedling stage under greenhouse conditions with artificial inoculation of *Uromyces vignae* isolate ZXL01, and at the adult stage under natural field infection at a single experimental site. Genome-wide association analysis (GWAS) was conducted separately for the two assessments and identified fifteen SNPs significantly associated with rust resistance. Ten SNPs were identified at the seedling stage on chromosomes 1 and 3, while the remaining five SNPs were detected at the adult stage on chromosome 9. The SNPs associated with adult-stage rust resistance were converted into Kompetitive Allele-Specific PCR (KASP) markers, which were subsequently validated in an independent panel. The validation confirmed that the resistance-associated alleles were predominantly detected in highly resistant accessions, suggesting that these markers have potential utility for the preliminary screening of rust-resistant germplasm, although further breeding validation is required.

## 1. Introduction

Adzuki bean [*Vigna angularis* (Willd.) Ohwi & Ohashi] is a leguminous crop belonging to the genus *Vigna* in the family Fabaceae, valued both as a food source and for its medicinal properties. It is currently cultivated in more than 30 countries worldwide, with major production areas in East Asia (China, Japan, and South Korea), as well as South and Southeast Asia [1]. As the center of origin and the largest producer of adzuki bean, China holds abundant germplasm resources and has developed numerous distinctive landraces and cultivars across the country [2]. Additionally, adzuki bean features a short growth cycle, broad adaptability, and nitrogen-fixing capacity. It is well suited for rotation or intercropping with major crops, thereby optimizing cropping systems and supporting sustainable agricultural development.

However, adzuki bean production is severely constrained by various diseases. Among these, rust, caused by the fungus *Uromyces vignae*, is one of the most significant diseases, adversely affecting both yield and quality. This disease occurs frequently in major adzuki bean-producing regions of China [3]. Under favourable environmental conditions, urediniospores of the rust fungus infect adzuki bean leaves and form numerous uredinia on the leaf surface, severely impairing photosynthesis. This leads to poor grain filling and substantial yield losses, reaching 20–60% in severe cases [4]. Therefore, the discovery and utilization of rust-resistance genes in adzuki bean have become an urgent priority for resistance breeding.

Currently, adzuki bean breeding for rust resistance relies predominantly on traditional phenotypic screening. This approach requires a full growing season for field evaluation, while greenhouse inoculation assays, though environmentally controllable, are labor-intensive and unsuitable for large-scale application [4]. Furthermore, rust disease severity is highly influenced by environmental conditions, and the uneven distribution of natural inoculum in the field often compromises the accuracy and reproducibility of phenotypic assessments.

Marker-assisted selection (MAS) offers a promising strategy to overcome certain limitations of conventional phenotypic screening. Its primary advantage is that marker genotypes can be identified without requiring phenotypic expression of the trait, reducing reliance on field evaluation and enabling selection at early growth stages. Notably, the recent availability of a high-quality adzuki bean reference genome [1], combined with rapid advances in high-throughput sequencing technologies, has made the large-scale development and application of molecular markers in adzuki bean breeding practically feasible.

In recent years, significant progress has been made in elucidating the molecular basis of rust resistance in adzuki bean. Resistant cultivars trigger immune recognition via pattern recognition receptors (PRRs), activate the expression of WRKY transcription factors and pathogenesis-related proteins, and enhance the activities of chitinase (CHI) and phenylalanine ammonia-lyase (PAL), thereby suppressing rust fungal colonization [4]. The N-acetyltransferase gene *VaNATA1* and the AP2/ERF transcription factor gene *VaDREB1A* have been cloned, with *VaNATA*1 acting as a positive regulator of rust resistance and *VaDREB1A* potentially participating in defense responses through the ethylene signaling pathway [5,6]. Liu et al. [7] identified nine NAC transcription factors induced by rust infection and responsive to methyl jasmonate treatment, suggesting that these genes may contribute to rust resistance via the jasmonic acid signaling pathway. To date, functional evidence has only been obtained for *VaNAC002* through transient overexpression in the tobacco–*Botrytis cinerea* system, while the roles of the other eight genes in rust resistance remain to be validated. Adzuki bean also produces defense proteins that directly inhibit fungal development. *VaEG45*, a plant natriuretic peptide, significantly suppresses urediniospore germination and mycelial growth of the rust fungus, with its mechanism associated with β-1,3-glucanase activity [8]. Collectively, these findings have provided initial insights into the molecular mechanisms underlying rust resistance in adzuki bean and laid a foundation for future discovery of resistance genes.

Since the release of the adzuki bean draft genome [9], genomic studies in this species have advanced considerably. Early efforts focused primarily on genetic linkage mapping; for example, a high-density map constructed via SLAF-seq was developed to identify flowering time QTLs [10], and another genetic map was built for seed size QTL analysis [11]. More recently, the rapid advancement of whole-genome resequencing (WGRS) has accelerated trait mapping in adzuki bean. A chromosome-level reference genome for the cultivar “Jingnong 6” was assembled, and 322 accessions were resequenced to identify domestication-related candidate genes via GWAS [1]. GWAS has similarly been used to detect genetic loci associated with leaf morphology [12] and seed iron accumulation [13], respectively. Notably, a high-quality genome of the elite cultivar ZH20 was assembled, and 546 diverse accessions were resequenced, identifying 251 loci significantly associated with eight key agronomic traits [14]. Despite these advances, genetic mapping of rust resistance genes in adzuki bean remains limited. To date, only one resistance gene, VaRust1, has been preliminarily mapped [15]. Collectively, these findings confirm that GWAS-based approaches are both feasible and effective for dissecting agronomically important traits in adzuki bean.

This study aimed to identify SNP loci associated with rust resistance in adzuki bean, develop practical molecular markers, and provide genetic resources and selection tools for marker-assisted resistance breeding. Its specific objectives were as follows: genotyping-by-sequencing (GBS) was performed to discover genome-wide SNPs and analyze the genetic structure of the adzuki bean panel; the rust resistance phenotypes of 178 accessions were evaluated under two environmental conditions; a genome-wide association study (GWAS) was conducted to detect SNP loci significantly associated with rust resistance; and significant SNPs were validated in an independent panel to screen for reliably confirmed candidate markers.

## 2. Results

### 2.1. GBS Sequencing of Adzuki Bean Accessions

Genotyping-by-sequencing (GBS) was performed on 178 adzuki bean accessions. The raw GBS sequencing data have been deposited in the Genome Sequence Archive (GSA) at the National Genomics Data Center (NGDC) under accession number CRA052398. After quality control and filtering of the raw sequencing data, a total of 855,255,382 high-quality clean reads were obtained, with an average of 4,804,805 reads per sample, indicating that the sequencing data were sufficient for subsequent analyses. The total sequencing data amounted to 124.87 Gb, with a mean of 0.70 Gb per sample. Quality assessment showed that the Q20 and Q30 values were 95.67% and 89.07%, respectively, demonstrating that the proportions of bases with accuracy exceeding 99% and 99.9% reached these thresholds, which confirms the high quality and reliability of the sequencing data. The clean reads were mapped to the adzuki bean reference genome (GCF_016808095.1), yielding an average mapping rate of 89.71%. This result indicates high homology between the sequencing data and the reference genome and further validates the applicability of the reference genome to the study panel (Table S1).

### 2.2. SNP Calling in Adzuki Bean

**Figure 1:**
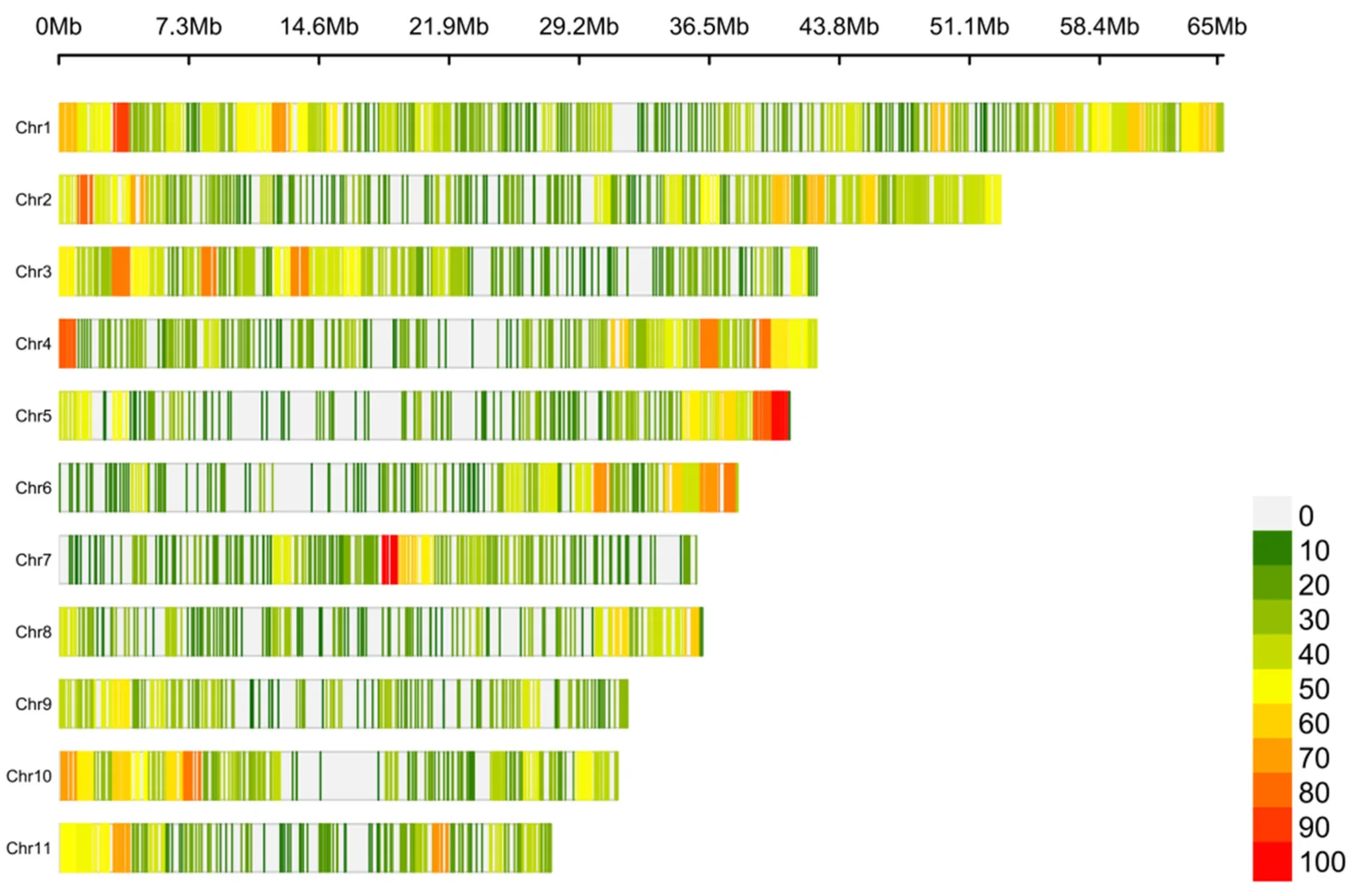
SNP density map for adzuki bean, generated using 1 Mb sliding windows. The x-axis indicates the physical position (in Mb) of markers on each chromosome, while the y-axis represents the 11 chromosomes (Chr1–Chr11). A color gradient ranging from dark green to dark red corresponds to the number of markers distributed in each chromosomal interval, with darker red indicating a higher marker count and darker green indicating a lower marker count.

After alignment to the adzuki bean reference genome and stringent quality control, a total of 12,153 high-quality SNP loci were obtained. SNP density varied considerably among chromosomes, ranging from 20.94 SNPs/Mb on chromosome 8 to 33.70 SNPs/Mb on chromosome 1, representing an approximately 1.6-fold difference. This variation may be attributable to differences in chromosome size, recombination rates, and centromeric regions. On average, approximately 1105 SNP loci were distributed per chromosome, with a genome-wide average SNP density of 26.56 SNPs/Mb (Figure 1), indicating that the GBS approach generated a sufficiently dense marker set for subsequent genome-wide association studies. Overall, the SNPs were relatively evenly distributed across the genome, although localized dense and sparse regions were also observed. Specifically, SNP-dense regions (with more than 80 markers per 1 Mb) were detected on chromosomes 1, 2, 3, 4, 5, and 7, which may correspond to gene-rich regions or recombination hotspots, whereas marker gaps were present on chromosomes 4, 5, 6, and 10, likely associated with centromeric regions or repeat-rich genomic areas.

### 2.3. Analysis of Population Structure and Genetic Relationships in the Adzuki Bean Species

**Figure 2:**
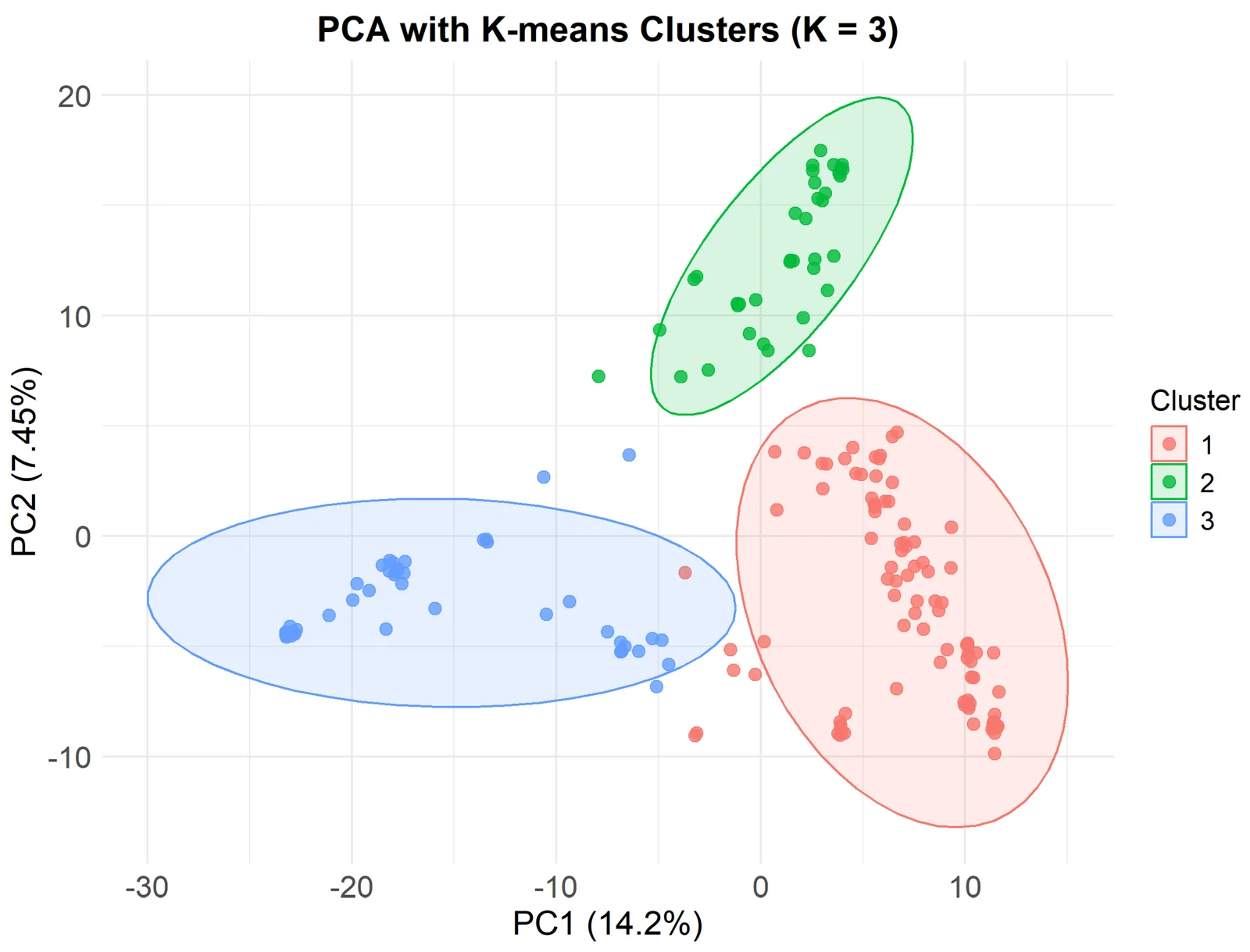
Principal component analysis of 178 adzuki bean accessions based on SNP markers. The x-axis represents the first principal component (PC1), and the y-axis represents the second principal component (PC2). Red, green, and blue dots represent samples from three distinct groups, respectively. The corresponding confidence ellipses for each group (red, green, and blue) show that the three groups are clearly distinguishable in the two-dimensional principal component space, indicating a robust clustering pattern based on the first two principal components.

Principal component analysis (PCA) was performed on 178 adzuki bean accessions using the identified SNP markers (Figure 2). The variance explained by PC1–PC5 is 14.20%, 7.45%, 3.88%, 3.54%, and 2.83%, respectively, with a cumulative contribution of 31.90%. The remaining 173 PCs each explain less than 2.3%, collectively accounting for 68.10% of the total variance. Next, K-means clustering was performed based on the first five principal components. When K was set to 3, the 178 accessions were divided into three distinct genetic clusters, and these three subpopulations were clearly separated in the PCA plot, indicating the presence of significant population stratification among the tested accessions. Of the three clusters, Cluster 1 included 96 accessions, Cluster 2 included 37 accessions, and Cluster 3 included 45 accessions.

**Figure 3:**
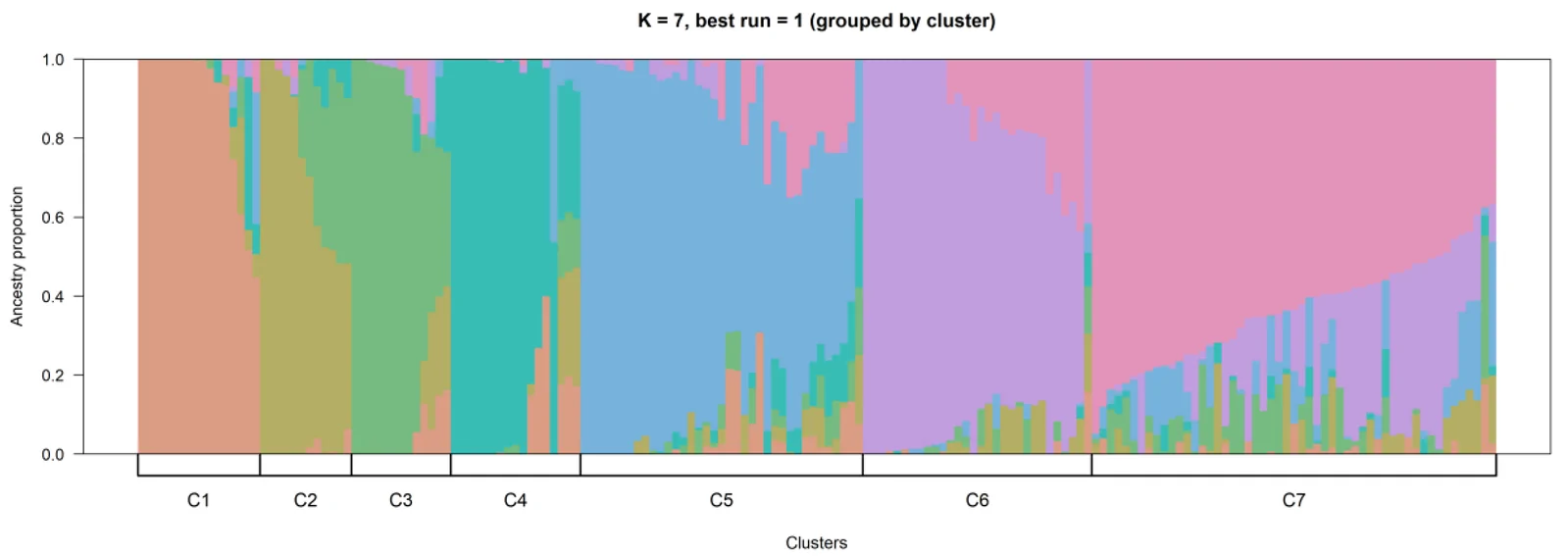
Population genetic structure analysis of 178 adzuki bean accessions. Each vertical bar represents an individual, with the proportion of each of the seven inferred ancestral genetic components (C1–C7) in its genome indicated by the colored segments of the bar. The ancestry proportion for each individual is represented by the height of its corresponding color segments.

To further analyze the population genetic structure of the 178 adzuki bean accessions, we inferred population structure using the LEA package (v3.10.2) in R. We calculated the cross-entropy criterion for different assumed numbers of ancestral populations (K values) to identify the optimal number of clusters. When K ranged from 1 to 15, the cross-entropy reached its minimum value of 0.41 at K = 7, indicating that the population is optimally divided into seven genetic subpopulations (C1–C7) (Figure 3). The number and proportion of accessions in each subpopulation are as follows: C1, 16 accessions (8.99%); C2, 12 accessions (6.74%); C3, 13 accessions (7.30%); C4, 17 accessions (9.55%); C5, 37 accessions (20.79%); C6, 30 accessions (16.85%); and C7, 53 accessions (29.78%). Significant differences in allele frequencies were observed among these subpopulations, confirming that the population harbors abundant genetic diversity.

**Figure 4:**
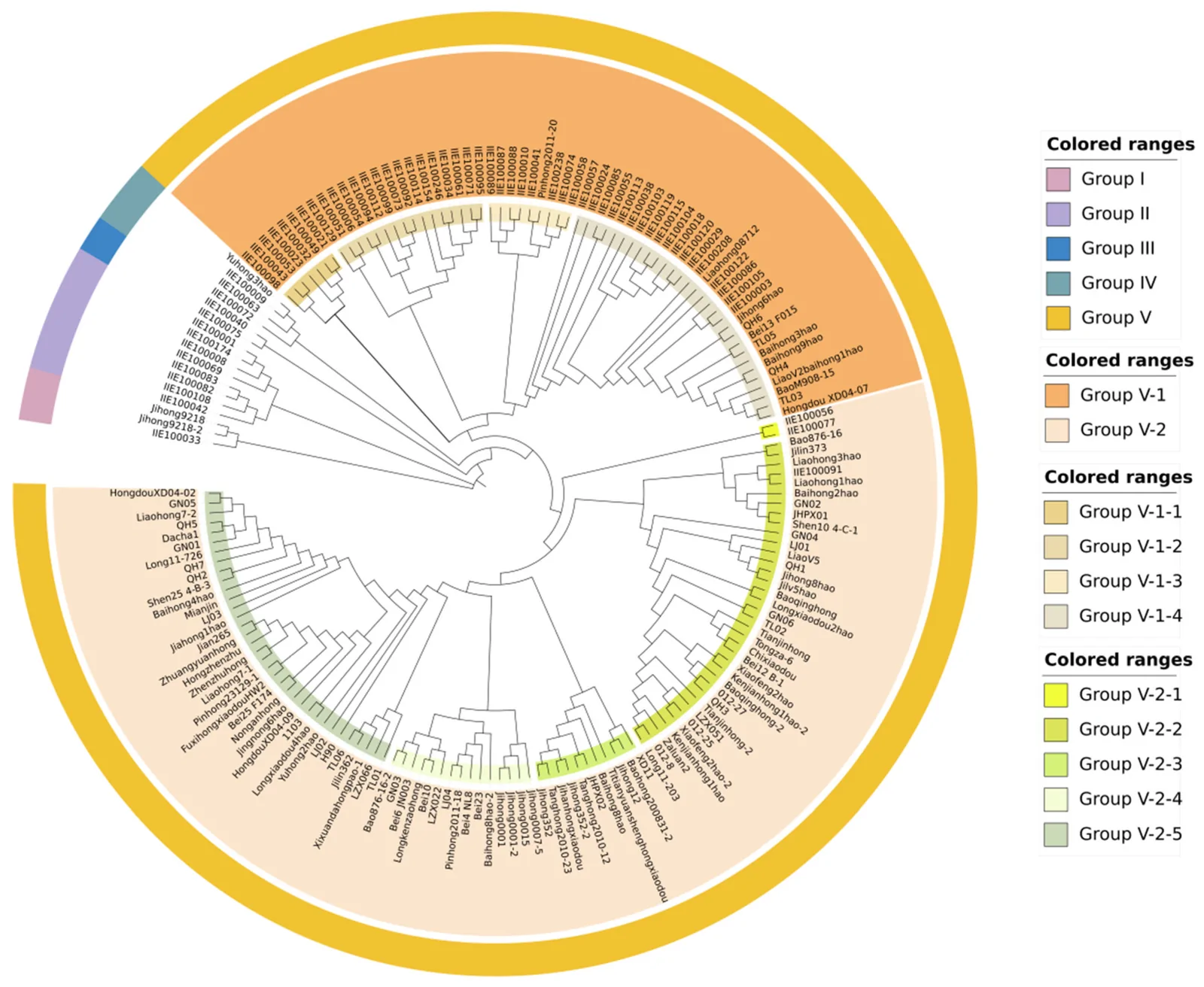
Cluster analysis diagram of 178 adzuki bean resources based on SNP markers. The phylogenetic tree was constructed using the Neighbor-Joining (NJ) method based on the Euclidean distance matrix. The 178 accessions were divided into five major groups. Groups I–IV contained 3, 8, 2, and 4 accessions, respectively, suggesting unique genetic backgrounds and potential breeding value. Group V contained the largest number of accessions and was further divided into two subgroups: V-1, mainly from Hebei; and V-2, mainly from Heilongjiang. Group V-2 could be further subdivided into five genetic clusters, indicating substantial genetic differentiation among Heilongjiang accessions.

Based on the Euclidean distance matrix, we constructed a phylogenetic tree using the Neighbor-Joining (NJ) method (Figure 4) to analyze genetic relationships among the tested adzuki bean accessions. The NJ tree showed that the 178 adzuki bean accessions can be divided into five major groups. Among these, Group I, Group II, Group III, and Group IV contain 3, 8, 2, and 4 accessions, respectively. The relatively small number of accessions in these four groups suggests that these materials likely have unique genetic backgrounds and hold potential value for the genetic improvement of adzuki bean. Group V contains the largest number of accessions and can be further divided into two subgroups: Group V-1 is mainly composed of accessions collected from Hebei Province, while Group V-2 primarily consists of accessions from Heilongjiang Province. Within Group V-2, accessions can be further divided into five genetic clusters, indicating that accessions from Heilongjiang have considerable genetic differentiation.

### 2.4. Rust Assessment in Adzuki Bean Accessions

**Figure 5:**
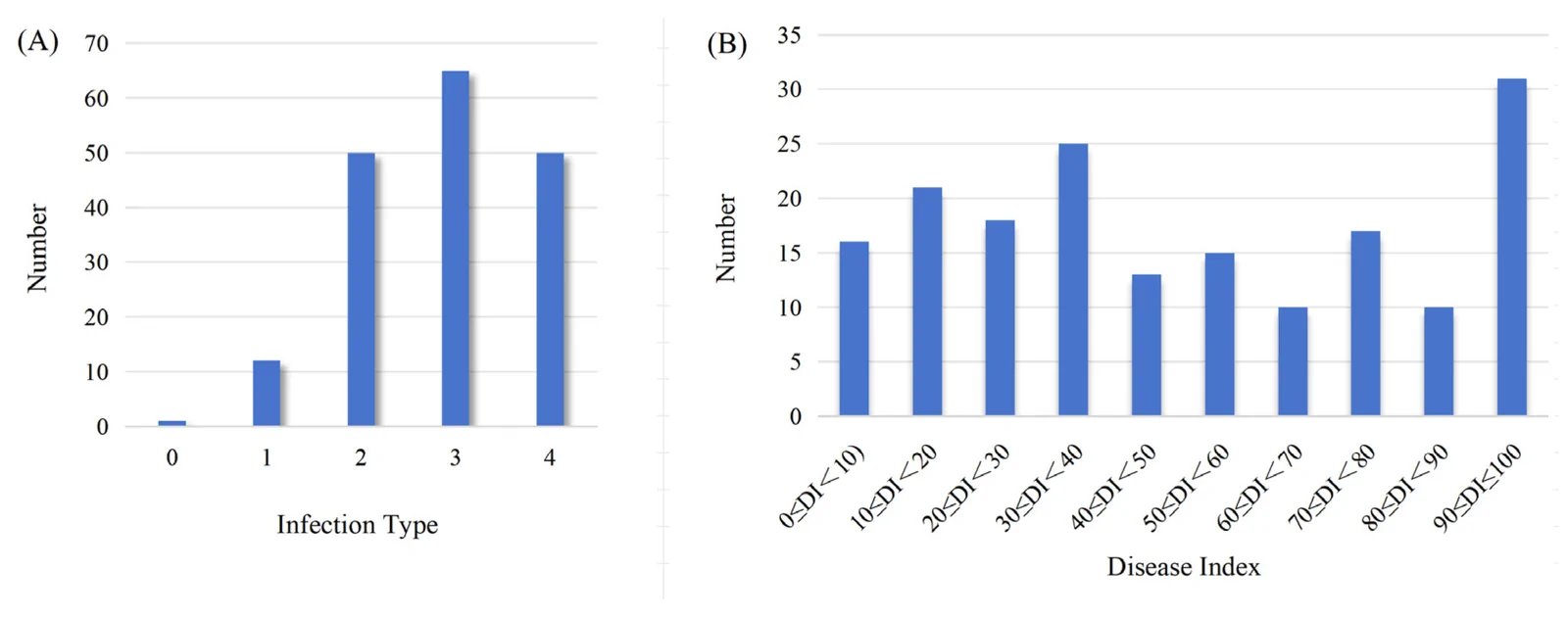
Evaluation of rust resistance in 178 adzuki bean accessions at the seedling and adult stages. (**A**) Frequency distribution of infection types at the seedling stage. The x-axis represents the infection type scale (0–4), and the y-axis denotes the number of accessions in each category. (**B**) Frequency distribution of disease index intervals under natural field conditions at the adult stage. The x-axis represents disease index intervals, and the y-axis indicates the number of accessions in each interval.

Evaluation of rust resistance at the seedling stage across the 178 adzuki bean accessions (Table S2) revealed a continuous distribution of infection types. Specifically, infection type “0” (highly resistant) was observed in only one accession (0.56%), while infection type “1” (resistant) and type “2” (moderately resistant) were recorded in 12 (6.74%) and 50 (28.09%) accessions, respectively. Infection type “3” (moderately susceptible) accounted for the largest proportion, with 65 accessions (36.52%), whereas infection type “4” (highly susceptible) was detected in 50 accessions (28.09%) (Figure 5A). These results indicate that the tested accessions exhibited diverse levels of rust resistance at the seedling stage, suggesting substantial genetic variation in resistance among different adzuki bean accessions.

Under natural field infection conditions, the disease index of the 178 accessions ranged from 0.00 to 100.00, indicating a broad continuum of disease responses ranging from complete resistance to full susceptibility (Figure 5B). Frequency distribution analysis further revealed the presence of both highly resistant and highly susceptible materials within the panel. Specifically, 16 accessions exhibited a disease index below 10, among which IIE100041 and IIE100099 showed a disease index of 0, representing complete resistance under field conditions (Table S2). These two accessions, displaying complete rust resistance under field conditions, represent valuable genetic resources for resistance breeding.

### 2.5. Association Analysis of Rust Resistance in Adzuki Bean

**Figure 6:**
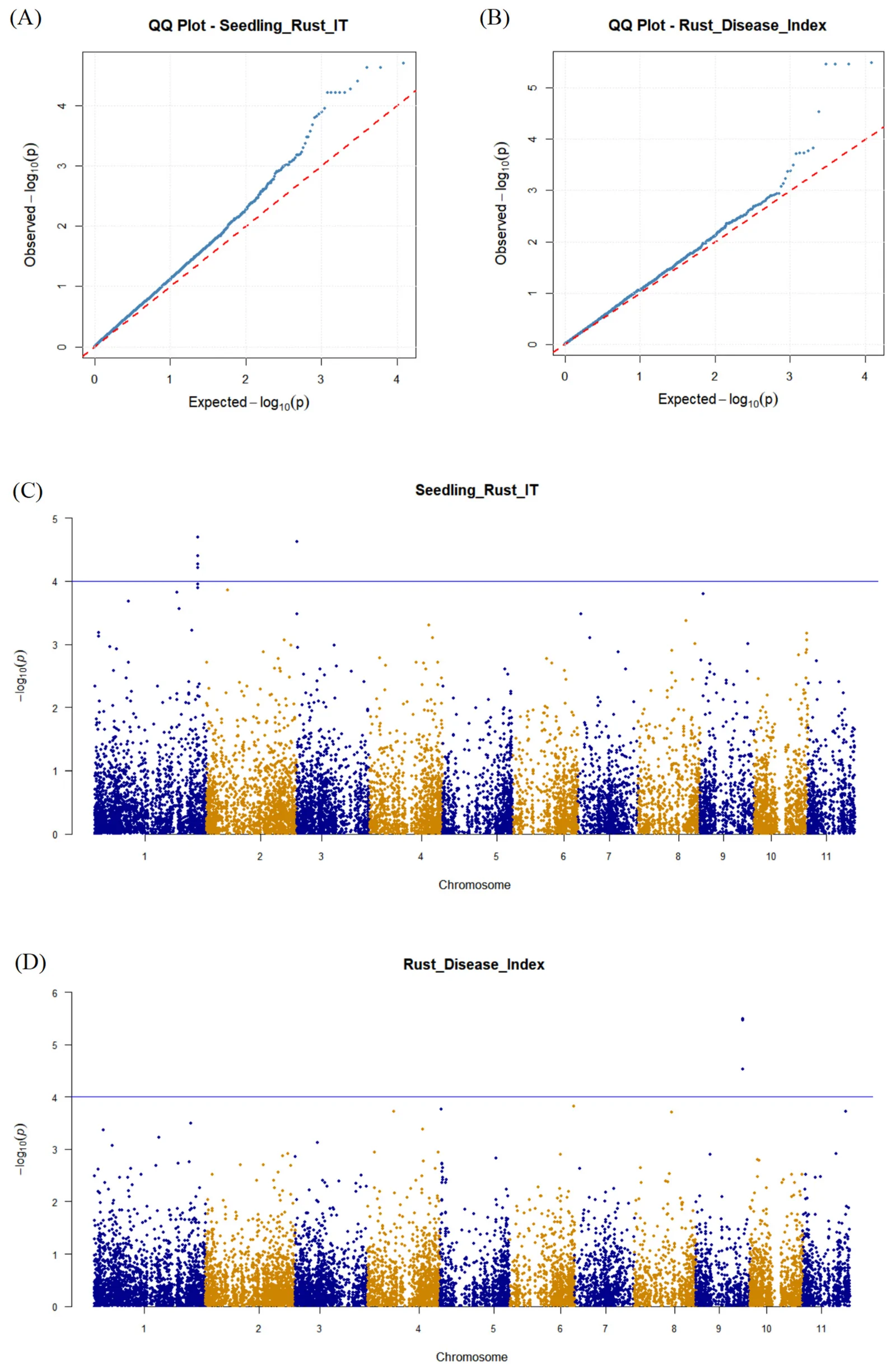
Quantile–quantile (QQ) plots and Manhattan plots of genome-wide association analysis for rust resistance loci in adzuki bean. (**A**,**B**) QQ plots for seedling rust resistance (Seedling_Rust_IT) and the rust disease index (Rust_Disease_Index). The red dashed line indicates the expected values under the null hypothesis, blue dots represent observed values, and upward deviation of the dot distribution from the expected line in the tail indicates significantly associated SNPs. (**C**,**D**) Manhattan plots for the two traits. The x-axis represents chromosomes (1–11), the y-axis represents −log_10_(*(p*), and the blue line indicates the significance threshold −log_10_(*p*) = 4.08. Ten SNPs were detected for seedling-stage resistance, and five for adult-stage resistance.

To identify genetic loci associated with rust resistance in adzuki bean, genome-wide association studies (GWAS) were performed using phenotypic data from both greenhouse seedling inoculation (infection types) and field adult-stage natural infection (Disease Index), combined with genome-wide SNP markers. We applied a threshold of *p* < 1/N = 1/12,153 = 8.23 × 10^−5^, corresponding to −log_10_(*p*) > 4.08. This threshold was selected to balance false positive control and statistical power, as GBS yields a moderate number of markers and the standard Bonferroni correction (0.05/N) may be overly stringent for detecting SNPs with moderate effects. The seedling-stage analysis identified ten SNPs significantly associated with rust resistance, eight on chromosome 1 and two on chromosome 3 (Table 1, Figure 6A,C). The adult-stage analysis detected five significant SNP markers, all located on chromosome 9 (Table 1, Figure 6B,D). Notably, the eight SNPs on chromosome 1 were clustered within a 100 bp window, indicating strong linkage disequilibrium (LD). No overlapping SNPs were detected between the seedling and adult stages, suggesting that rust resistance at these two stages may be governed by distinct genetic loci. These associated genomic regions represent promising targets for future fine mapping and candidate gene discovery.

### 2.6. Application of Rust Resistance-Associated Loci in Adzuki Bean

To further validate the reliability of the association results and develop practical molecular markers, we selected five SNP loci on chromosome 9 that were significantly associated with adult-stage rust resistance and converted them into Kompetitive Allele-Specific PCR (KASP) markers (Table 2).

Based on the preliminary resistance evaluation, 69 adzuki bean accessions with continuous phenotypic distribution were randomly selected from the natural population for genetic analysis. To assess the association between the genotypes of significant SNPs and adult-stage phenotypes, we jointly analyzed the five significant SNPs on chromosome 9 with the disease indices of these 69 accessions. The results showed that these five SNPs formed two major haplotypes. Specifically, four accessions carrying the C-T-T-A-A mutant haplotype (IIE100041, IIE100103, IIE100074, and IIE100058) exhibited very low disease indices (0–3.18), corresponding to immune or highly resistant phenotypes. In contrast, the vast majority of accessions carrying the A-G-A-C-G haplotype showed a wide range of disease indices (5.96–100.00), encompassing phenotypes from highly resistant to highly susceptible (Table 3). Notably, among the accessions carrying the A-G-A-C-G haplotype, only a few (e.g., Liaohong1hao and Liaohong3hao) had low disease indices (<10).

## 3. Discussion

Sequencing is a core technological approach for revealing the fundamental genetic information of a species. Compared with whole-genome sequencing, genotyping-by-sequencing (GBS) is relatively cost-effective and well suited for large-scale population genetic studies. In this study, a total of 12,153 high-quality SNP markers were obtained in adzuki bean through GBS, with an average genome-wide density of 26.56 SNPs/Mb, a marker number sufficient to support effective genome-wide association studies. GBS has also been successfully applied in adzuki bean. A GBS analysis was conducted on 366 landraces originating from Korea, China, and Japan, yielding 6586 SNPs, and population structure analysis divided these landraces into three subpopulations [16]. These studies demonstrate that GBS can generate substantial numbers of molecular markers in adzuki bean and be effectively utilized for genetic diversity assessment and association analysis.

In recent years, genomic resources for adzuki bean have been steadily improved. A high-quality, chromosome-level genome assembly of adzuki bean was reported, spanning 447.8 Mb and covering 96.32% of the estimated genome, with contig and scaffold N50 values of 16.5 Mb and 41.0 Mb, respectively [17]. More than 98.2% of the 1614 BUSCO genes were fully recovered, and 25,939 genes were annotated, of which 98.23% received functional assignments. Given the continuously declining sequencing costs, whole-genome resequencing has become increasingly feasible and provides access to substantially larger marker sets. Indeed, using a combination of second- and third-generation sequencing with Hi-C-based chromosome scaffolding, the genome of the adzuki bean cultivar “Jingnong 6” was successfully assembled, and 322 accessions were resequenced, identifying 1,352,825 SNPs [1]. Furthermore, 546 accessions were resequenced, identifying 251 loci for key agronomic traits and demonstrating the superior resolution of WGRS [14]. Compared with WGRS, the GBS approach used in the present study yields lower marker density. However, GBS was sufficient to identify a candidate genomic region on chromosome 9 and develop KASP markers for preliminary breeding. In the future, higher-resolution WGRS-based GWAS will be essential to precisely pinpoint causal genes and further validate these SNPs.

Based on population structure analysis with K = 7, the 178 adzuki bean accessions were partitioned into seven genetic subpopulations (C1–C7). As shown in the structure plot, most individuals exhibited a relatively homogeneous genetic background, with their genomes predominantly derived from a single subpopulation, indicating a largely pure ancestry consistent with the self-pollinating nature of adzuki bean. Meanwhile, a small number of individuals showed admixed ancestry, suggesting possible gene flow among subpopulations. These results, combined with PCA-based K-means clustering (K = 3), revealed a hierarchical genetic structure within the population. PCA grouped the accessions into three main clusters, while the K = 7 population structure analysis further resolved fine-scale differentiation within these clusters, indicating that the tested materials harbor substantial genetic diversity.

Rust disease poses a serious threat to adzuki bean production, making the identification of rust resistance loci or genes highly valuable for accelerating the development of resistant cultivars through marker-assisted selection (MAS). In this study, we performed genome-wide association analysis (GWAS) using high-quality SNP markers obtained from genotyping-by-sequencing and detected a total of fifteen significantly associated SNPs. Among them, ten SNPs were identified at the seedling stage and distributed on chromosomes 1 and 3, whereas the five SNPs detected at the adult stage were all located on chromosome 9, with no overlap between the two stages.

The non-overlapping loci detected at the two stages suggest that distinct genetic factors may underlie resistance at the seedling and adult stages. However, as the two assessments also differed in growing environment, infection conditions, and phenotypic measurement protocols, this study cannot disentangle the effects of developmental stage from those of assessment conditions. The stage-specific resistance hypothesis therefore remains a possibility requiring further validation under controlled conditions. Although our study cannot disentangle these factors, stage-specific resistance is a well-documented phenomenon in other crop-pathogen interactions. For example, both stripe rust and powdery mildew in wheat exhibit clear differentiation between seedling resistance and adult-plant resistance [18,19]. Additionally, differences in infection conditions, host physiological status, and defense response mechanisms between the seedling and adult stages may collectively contribute to the differential expression of resistance loci across growth stages. These results suggest that evaluating germplasm at both stages may help identify a broader spectrum of resistance loci. However, as resistance durability and stability were not assessed in this study, whether combining seedling and adult-stage resistance would enhance field durability remains to be tested in future breeding programs.

Adult-stage association analysis detected five significantly associated SNPs on chromosome 9, suggesting that this chromosome may harbor important genomic regions conferring rust resistance in adzuki bean. After converting these SNPs into KASP markers, genotyping was performed in a validation panel of 69 accessions. The KASP markers developed here can be regarded as preliminarily validated candidate markers linked to the chromosome 9 genomic region. Their utility was verified in only four highly resistant accessions under a single field environment; therefore, their predictive reliability across broader genetic backgrounds and different infection conditions remains to be established. Furthermore, because the five SNPs lie within an approximately 35 kb interval, they likely represent a single association region or haplotype block rather than five independent loci. The KASP markers therefore tag this candidate region and do not by themselves identify the causal gene(s). The fact that this region is located on a different chromosome from the previously reported *VaRust1* (chromosome 3) [15] suggests that it may represent a distinct rust resistance-associated genomic region, although this requires further confirmation.

To confirm the novelty of the loci identified in this study, we performed biological and positional comparisons between our loci and previously reported rust resistance factors, with a specific focus on the cowpea *QTL Ruv1* [20] and the adzuki bean gene *VaRust1* [15]. The *Uromyces vignae* isolate ZXL01 used in this study exhibits host specificity, as it is non-pathogenic to cowpea [21], indicating distinct resistance mechanisms between the two species. Additionally, the previously mapped *VaRust1* gene is located on Chr3 (20.46–26.20 Mb). While our two seedling-resistance SNPs are also on Chr3, their physical positions (31,982 bp and 54,870 bp) lie more than 20 Mb away from the *VaRust1* interval. These comparisons provide strong evidence that the loci identified in this study are novel and independent of previously reported resistance factors.

Although this study successfully identified rust resistance-associated SNPs and developed preliminary KASP markers, several limitations must be acknowledged. First, seedling inoculation relied on a single *Uromyces vignae* isolate (ZXL01). Given the potential pathogenic variability of the rust fungus, future studies should validate these loci using multiple isolates. Second, the adult-stage GWAS was conducted in a single field environment. Although susceptible controls developed uniform symptoms and KASP markers were preliminarily validated, the lack of multi-year, multi-location phenotypic data remains a limitation. Future trials across multiple environments are needed to further verify the stability and robustness of these loci. Third, the marker density generated by GBS is relatively lower than that of whole-genome resequencing (WGRS). While GBS was sufficient for identifying candidate regions in this study, future research using WGRS or high-density arrays will be required to achieve finer mapping and pinpoint the causal genes underlying rust resistance in adzuki bean.

## 4. Materials and Methods

### 4.1. Plant Materials and Fungal Isolate

A total of 178 adzuki bean accessions were used in this study, and isolate ZXL01 of *Uromyces vignae* was used for inoculation. All tested accessions and the rust isolate were maintained at the National Coarse Cereals Engineering Research Center.

### 4.2. Greenhouse and Field Cultivation of Adzuki Bean

Rust resistance evaluation of the tested adzuki bean accessions was conducted at the seedling stage in the greenhouse in 2021 and at the adult stage in the field in 2022. For seedling-stage assays, plastic pots (18 cm in diameter) were used, with eight seeds sown per pot. After emergence, seedlings were thinned to five uniform and healthy plants per pot and grown under standard management conditions in a greenhouse maintained at approximately 25 °C. For adult-stage evaluation, the trial was carried out in the experimental field of Heilongjiang Bayi Agricultural University. The field site had uniform soil fertility, well-established irrigation and drainage facilities, and a history of natural rust occurrence over multiple years. Each accession was planted in rows of 2 m length. Conventional field management practices, including irrigation, fertilization, and weed control, were applied. To ensure sufficient and consistent disease pressure, the susceptible cultivar ‘Baoqinghong’ was planted at both ends and in the middle of the experimental plot as a susceptible control.

### 4.3. Genotyping-by-Sequencing and Quality Control

After rust disease assessment, five representative plants were selected from each accession, and one leaf was collected from each plant. The five leaves from the same accession were pooled into a single sample for genomic DNA extraction. The collected leaf samples were immediately frozen in liquid nitrogen and stored at −80 °C for later use. Genotyping-by-sequencing (GBS) was performed by Shanghai Ouyi Biomedical Technology Co., Ltd. (Shanghai, China), following standard procedures for library construction and high-throughput sequencing. Genomic DNA was digested with the restriction enzymes *PstI*-HF/*MspI* to construct GBS libraries. Sequencing was performed on the Illumina NovaSeq platform with 150 bp paired-end (PE150) reads. Raw reads were quality-filtered using fastp to remove adapter sequences and low-quality bases. Clean reads were aligned to the adzuki bean reference genome (GCF_016808095.1) using BWA-MEM (v0.7.17) software, and SNPs were called using GATK4 HaplotypeCaller (v4.1.3.0) software [22]. To reduce variant detection error, only sites supported by reads with a sequencing depth of at least 4 were retained [23], as this threshold also balances genotype accuracy. On this basis, SNPs with a missing rate greater than 20% and a minor allele frequency (MAF) lower than 0.01 were further filtered out. This step ensured the accuracy and representativeness of markers for subsequent population genetic analyses and genome-wide association studies, ultimately yielding a high-quality SNP dataset for downstream analyses.

### 4.4. Population Genetic Structure and Cluster Analysis of Adzuki Bean Accessions

Based on the high-quality SNP markers, population genetic structure and clustering analyses were performed. Genotype data were imported using the readxl package in R software (v4.6.1), and a genind object was subsequently constructed with the adegenet (v2.1.11) package to facilitate downstream population genetic analyses. Principal component analysis (PCA) was applied for dimensionality reduction to assess the primary sources of genetic variation among populations, followed by K-means clustering to preliminarily resolve population structure. Scatter plots with 95% confidence ellipses were generated using ggplot2 to visualize subpopulation differentiation. To further resolve the population genetic structure with higher precision, ancestry component analysis was carried out using the LEA package. Cross-entropy criterion under different assumed numbers of ancestral populations (K values) was computed to determine the optimal number of clusters, yielding a reliable population partition. In addition, a phylogenetic tree was constructed using the neighbor-joining (NJ) method based on a Euclidean distance matrix to evaluate genetic relationships among individuals, serving as a complementary validation of the population structure results.

### 4.5. Rust Resistance Evaluation in Adzuki Bean

Under greenhouse conditions, inoculation was performed when the first true leaves of the tested plants fully expanded, using the adzuki bean *Uromyces vignae* isolate ZXL01 at a concentration of 1 × 10^4^ urediniospores/mL. The spore suspension was prepared with sterile water and evenly sprayed onto leaf surfaces via the spray inoculation method. After inoculation, the plants were kept at 18 °C under high humidity for 24 h, then transferred to a greenhouse maintained at approximately 22 °C, 90% relative humidity, with a 12 h light/12 h dark cycle. At 12 days post-inoculation, once the susceptible control cultivar “Baoqinghong” developed sufficient and uniform disease symptoms, the rust resistance level of each tested accession was evaluated. Five representative plants from each accession were sampled as biological replicates, and the evaluation was performed using a 0–4 rating scale. “0”, no visible uredinia (highly resistant); “1”, 1–5 uredinia per 1.5 cm^2^ leaf (resistant); “2”, 6–19 uredinia per 1.5 cm^2^ leaf (moderately resistant); “3”, 20–39 uredinia per 1.5 cm^2^ leaf (moderately susceptible); 4, ≥40 uredinia per 1.5 cm^2^ leaf (highly susceptible). The mean infection type across biological replicates was calculated and used as the phenotypic value for each accession in the GWAS.

Field trials were conducted in the experimental field of Heilongjiang Bayi Agricultural University, a site with a history of annual natural rust occurrence. After susceptible controls planted at both ends and in the middle of each plot had developed full symptoms, five representative plants per accession were randomly selected, and 20 leaves were collected from each plant. Infection types were recorded in detail, and the disease index was subsequently calculated from the survey data to assess the resistance level of each accession under natural field conditions.

The calculation formula for the disease index (DI) is as follows:

DI = Σ(n*_i_*× s*_i_*)/(N × 4) × 100,

n_i_ is the number of leaves with disease score i.

s_i_ is the disease score.

N is the total number of leaves surveyed.

### 4.6. Genome-Wide Association of Rust Resistance Loci in Adzuki Bean

Using single nucleotide polymorphism (SNP) markers, combined with seedling rust resistance scores and adult-stage disease indices, a genome-wide association study (GWAS) was conducted to identify genetic loci tightly associated with rust resistance in adzuki bean. The GWAS was performed using the mixed linear model (MLM) implemented in TASSEL 5 software (v5.2.73). In the analysis, SNP markers were fitted as fixed effects. To control for population stratification and relatedness, the top 5 principal components (PCs) and a kinship (K) matrix were included in the MLM as fixed and random effects, respectively. The genome-wide significance threshold was set at *p* < 1/12,153 = 8.23 × 10^−5^, corresponding to −log_10_(*p*) > 4.08.

### 4.7. Development and Validation of KASP Markers

The validation panel, consisting of 69 adzuki bean accessions, exhibited a continuous phenotypic distribution for disease index (DI) ranging from 0 to 100. Genomic DNA was extracted from young leaves of these accessions, and the five significant SNPs were genotyped using KASP assays. The association between genotypes and DI was evaluated by comparing phenotypic distributions across different genotypic groups. Based on the 300 bp flanking sequences of each associated SNP, two allele-specific forward primers and one common reverse primer were designed using Primer3 (v2.6.1) software. The 3′ ends of the allele-specific primers matched their respective alleles, while their 5′ ends carried distinct tail sequences complementary to universal FAM and HEX fluorescent probes. PCR reactions were performed in a 10 μL volume containing 1 μL of genomic DNA (20 ng/μL), 1.4 μL of KASP primer mix (10 μmol/L), and 5 μL of 2× KASP Master Mix, with ddH_2_O added to reach the final volume. The thermal cycling program was as follows: initial denaturation at 94 °C for 15 min; 10 cycles of denaturation at 94 °C for 20 s and annealing at 61 °C for 60 s (decreasing by 0.6 °C per cycle), followed by 35 cycles of denaturation at 94 °C for 20 s and annealing at 55 °C for 60 s. After amplification, FAM and HEX fluorescence signals were detected using a Bio-Rad CFX96 real-time PCR system, and genotyping was performed with Bio-Rad CFX Manager 3.0 software.

## 5. Conclusions

In this study, genotyping-by-sequencing (GBS) was used to identify genome-wide SNPs in 178 adzuki bean accessions, yielding 12,153 high-quality SNPs and enabling characterization of population genetic structure (three clusters by PCA, seven subpopulations at K = 7). Based on two phenotype datasets, seedling-stage greenhouse inoculation and adult-stage natural field infection, GWAS identified 15 SNPs significantly associated with rust resistance: 10 at the seedling stage (on chromosomes 1 and 3) and 5 at the adult stage (chromosome 9), with no overlapping SNPs between the two stages. The five adult-stage SNPs on chromosome 9 were converted into KASP markers and validated in 69 accessions; four accessions carrying the C–T–T–A–A haplotype showed very low disease index (0–3.18), corresponding to immune or highly resistant phenotypes. This chromosome 9 region differs from the previously reported *VaRust1* locus (chromosome 3) and may represent a novel rust resistance-associated region. This study provides candidate genomic regions and potentially useful KASP markers for marker–assisted breeding of rust–resistant adzuki bean, although the predictive reliability of these markers still requires further validation across broader genetic backgrounds and multiple environments and isolates.

## Figures and Tables

**Table 1:** Significant SNPs associated with rust resistance in adzuki bean.

Growth Stage	SNP	Chr.	Position	Alleles (Ref/Alt)	*p*-Value	Estimated Effect
Seedling-stage	*Vig8560299336*	Chr.1	60299336	C/G	2.01 × 10^−5^	−0.982
	*Vig8731982*	Chr.3	31982	G/C	2.38 × 10^−5^	−1.000
	*Vig8754870*	Chr.3	54870	A/G	2.38 × 10^−5^	−1.000
	*Vig8560299427*	Chr.1	60299427	A/C	3.96 × 10^−5^	−1.316
	*Vig8560299385*	Chr.1	60299385	C/T	5.37 × 10^−5^	−0.903
	*Vig8560299346*	Chr.1	60299346	A/C	6.21 × 10^−5^	−0.894
	*Vig8560299357*	Chr.1	60299357	C/G	6.21 × 10^−5^	−0.894
	*Vig8560299358*	Chr.1	60299358	A/C	6.21 × 10^−5^	−0.894
	*Vig8560299374*	Chr.1	60299374	T/A	6.21 × 10^−5^	−0.894
	*Vig8560299382*	Chr.1	60299382	G/A	6.21 × 10^−5^	−0.894
Adult-stage	*Vig9327901413*	Chr.9	27901413	A/G	3.25 × 10^−6^	−64.197
	*Vig9327865995*	Chr.9	27865995	G/A	3.41 × 10^−6^	−63.965
	*Vig9327866047*	Chr.9	27866047	G/T	3.41 × 10^−6^	−63.965
	*Vig9327866058*	Chr.9	27866058	A/T	3.41 × 10^−6^	−63.965
	*Vig9327900770*	Chr.9	27900770	C/A	2.91 × 10^−5^	−65.597

Note: Ref, reference allele; Alt, alternative allele. The estimated effect is calculated based on the Alt allele.

**Table 2:** Primer sequences of KASP markers associated with rust resistance in adzuki bean.

SNP	Primer 1-FAM (5′-3′)	Primer 2-HEX (5′-3′)	Common Primer C (5′-3′)
*Vig9327865995*	GAAGGTGACCAAGTTCATGCTCGTGCTCAAGAAAGCTGCAAA	GAAGGTCGGAGTCAACGGATTCGTGCTCAAGAAAGCTGCAAG	CGTCTCAAACCCTGTGATGTACCT
*Vig9327866047*	GAAGGTGACCAAGTTCATGCTAGGTACATCACAGGGTTTGAGAC	GAAGGTCGGAGTCAACGGATTCAGGTACATCACAGGGTTTGAGACT	ACTGCAGTATTAAGCTTAAAACTTGATGT
*Vig9327866058*	GAAGGTGACCAAGTTCATGCTCACAGGGTTTGAGACGTAATACATCAA	GAAGGTCGGAGTCAACGGATTCACAGGGTTTGAGACGTAATACATCAT	ATTAACAACTTCATTCAATTCAAAATTTG
*Vig9327900770*	GAAGGTGACCAAGTTCATGCTATGTGTCAAGTTTACAACCGGGATC	GAAGGTCGGAGTCAACGGATTATGTGTCAAGTTTACAACCGGGATA	TATAATCTTTTAAAAGATCCAAAACAAAAAC
*Vig9327901413*	GAAGGTGACCAAGTTCATGCTTTTTTCCATCCTTTCCTTATTTTTCTC	GAAGGTCGGAGTCAACGGATTTTTTTCCATCCTTTCCTTATTTTTCTT	GGAAACACAATGTGAGCAGTGAG

**Table 3:** KASP genotyping of 69 Adzuki bean resources and their rust disease index.

Accession	Rust Disease Index	Genotype of SNPs				
		*Vig9327865995*	*Vig9327866047*	*Vig9327866058*	*Vig9327900770*	*Vig9327901413*
Bei6 JN003	25.56	AA	GG	AA	CC	GG
Longkenzaohong	14.60	AA	GG	AA	CC	GG
TL05	100.00	AA	GG	AA	CC	GG
Bei13 F015	100.00	AA	GG	AA	CC	GG
QH1	46.72	AA	GG	AT	CA	GG
QH4	100.00	AA	GG	AA	CC	GG
QH7	37.06	AA	GG	AA	CC	GG
LJ01	21.09	AA	GG	AA	CC	GG
LJ02	36.04	AA	GG	AA	CC	GG
LJ03	25.41	AA	GG	AA	CC	GG
GN01	27.46	AA	GG	AA	CC	GG
Longxiaodou2hao	36.16	AA	GG	AA	CC	GG
GN04	61.28	AA	GG	AA	CC	GG
Jilin362	29.69	AA	GG	AA	CC	GG
Jilv5hao	75.64	AA	GG	AA	CC	GG
XD11	100.00	AA	GG	AA	CC	GG
LZX022	41.72	AA	GG	AA	CC	GG
LZX051	58.59	AA	GG	AA	CC	GG
H90	40.09	AA	GG	AA	CC	GG
Baoqinghong	100.00	AA	GG	AA	CC	GG
Dacha1	30.53	AA	GG	AA	CC	GG
Kenjianhong1hao-1	100.00	AA	GG	AA	CC	GG
Kenjianhong1hao-2	59.68	AA	GG	AA	CC	GG
Mianjin	10.82	AA	GG	AA	CC	GG
Nonganhong	18.48	AA	GG	AA	CC	GG
Zaluan2	71.82	AA	GG	AA	CC	GG
Jihong352	24.37	AA	GG	AA	CC	GG
Baoqinghong-2	92.82	AA	GG	AA	CC	GG
Zhenzhuhong	10.91	AA	GG	AA	CC	GG
Baihong8hao	53.00	AA	GG	AA	CC	GG
Baihong3hao	100.00	AA	GG	AA	CC	GG
Tianjinhong	33.13	AA	GG	AA	CC	GG
Jihong0001	14.77	AA	GG	AA	CC	GG
Liaohong7	37.11	AA	GG	AA	CC	GG
Liaohong1hao	6.94	AA	GG	AA	CC	GG
Liaohong3hao	5.96	AA	GG	AA	CC	GG
Pinhong2011-18	26.11	AA	GG	AA	CC	GG
Jihong0001-2	9.09	AA	GG	AA	CC	GG
Baihong8hao-2	43.33	AA	GG	AA	CC	GG
Tongza-6	50.30	AA	GG	AA	CC	GG
Liaohong08712	14.44	AA	GG	AA	CC	GG
Baohong200831-2	19.10	AA	GG	AA	CC	GG
Tanghong2010-23	100.00	AA	GG	AA	CC	GG
Tanghong2010-12	100.00	AA	GG	AA	CC	GG
012-27	42.98	AA	GG	AA	CC	GG
JHPX01	14.65	AA	GG	AA	CC	GG
Yuhong2hao	21.90	AA	GG	AA	CC	GG
Yuhong3hao	76.29	AA	GG	AA	CC	GG
IIE100058	3.18	CC	TT	TT	AA	AA
IIE100103	0.20	CC	TT	TT	AA	AA
IIE100074	1.87	CC	TT	TT	AA	AA
Zhuangyuanhong	13.97	AA	GG	AA	CC	GG
Jiahong1hao	68.10	AA	GG	AA	CC	GG
Jingnong6hao	36.66	AA	GG	AA	CC	GG
Tianjinhong-2	48.74	AA	GG	AA	CC	GG
Baihong2hao	54.35	AA	GG	AA	CC	GG
012-8	47.78	AA	GG	AA	CC	GG
012-25	100.00	AA	GG	AA	CC	GG
Chixiaodou	100.00	AA	GG	AA	CC	GG
Longxiaodou4hao	21.31	AA	GG	AA	CC	GG
IIE100003	92.44	AA	GG	AA	CC	GG
IIE100006	34.44	AA	GG	AA	CC	GG
IIE100038	20.39	AA	GG	AA	CC	GG
IIE100041	0.00	CC	TT	TT	AA	AA
IIE100042	39.20	AA	GG	AA	CC	GG
IIE100049	65.66	AA	GG	AA	CC	GG
IIE100051	30.93	AA	GG	AA	CC	GG
IIE100055	78.16	AA	GG	AA	CC	GG
IIE100057	60.29	AA	GG	AA	CC	GG

## Data Availability

The data presented in this study are available in this article. The raw GBS sequencing data have been deposited in the GSA at NGDC under accession number CRA052398 and will be publicly released two years after deposition. Before release, requests for special access should be directed to the corresponding author or the first author.

## Supplementary Materials

The following supporting information can be downloaded at: https://www.mdpi.com/article/10.3390/plants15213449/s1, Table S1: GBS sequencing of 178 adzuki bean accessions; Table S2: Rust resistance evaluation of adzuki bean accessions at seedling and adult stages.
