# Supplementary material for: Identifying Genes Conferring Rust Resistance in Adzuki Bean (Vigna angularis) Through Genome-Wide Association Analysis Based on Genotyping-by-Sequencing (GBS)
Source: Plants (Basel). 2026 Nov 5;15(21):3449. doi: 10.3390/plants15213449 (PMC13766009; doi:10.3390/plants15213449)
Supplement: Supplementary file 1 [file plants-15-03449-s001.zip › Table S1 _Table S2 .pdf]

Table S1 GBS sequencing of 178 adzuki bean accessions

Samples	Clean Reads	Clean Base(G)	Clean Base Percent	GC Content	>Q20	>Q30
TL01	4961612	0.72	97.97%	40.73%	95.80%	89.31%
Jilin373	4596022	0.67	97.64%	41.87%	95.37%	88.50%
Bao876-16	4981246	0.73	97.98%	41.15%	95.89%	89.49%
Hongdou XD04-07	4385592	0.64	97.81%	41.54%	95.32%	88.36%
TL02	5152562	0.75	98.00%	40.68%	95.79%	89.32%
TL03	4800196	0.7	97.66%	42.65%	95.81%	89.36%
Jian265	4582148	0.67	98.03%	40.63%	95.96%	89.65%
LZX066	4654854	0.68	97.67%	42.53%	95.69%	89.06%
LiaoV5	5471154	0.8	97.50%	42.17%	96.04%	89.80%
QH6	4498054	0.66	97.35%	44.66%	95.71%	89.14%
Jihanhongxiaodou	5187948	0.76	98.18%	40.42%	96.10%	89.92%
Bei6 JN003	5016562	0.73	97.74%	40.99%	95.81%	89.32%
Longkenzaohong	4932156	0.72	98.04%	41.11%	95.77%	89.29%
TL05	5280686	0.77	98.05%	40.56%	96.15%	89.96%
TL06	4625184	0.68	98.33%	40.76%	95.88%	89.48%
Jihong6hao	4989050	0.73	96.95%	43.67%	96.02%	89.75%
Bei13 F015	5230494	0.76	97.40%	42.35%	96.13%	89.98%
QH1	4891222	0.71	97.93%	41.40%	95.57%	88.87%
QH3	5230768	0.76	98.26%	40.20%	95.94%	89.59%
QH4	5058318	0.74	97.82%	42.31%	96.03%	89.79%
QH7	4363416	0.64	97.67%	41.33%	95.79%	89.31%
LJ01	4694504	0.69	97.91%	40.76%	96.05%	89.82%
QH5	5350172	0.78	98.11%	41.19%	95.72%	89.15%
QH2	4795562	0.7	97.62%	41.15%	95.85%	89.44%
LJ02	4815942	0.7	98.25%	40.78%	95.93%	89.56%
HongdouXD04-02	4668580	0.68	98.26%	41.26%	95.92%	89.56%
LJ03	4678992	0.68	97.56%	40.73%	96.27%	90.25%
GN01	4962836	0.72	97.67%	42.45%	96.08%	89.87%
GN02	4301020	0.63	95.95%	42.65%	96.00%	89.78%
HongdouXD04-09	4887108	0.71	97.78%	40.64%	96.18%	90.08%
Pinhong23129-1	4189854	0.61	97.58%	41.60%	96.16%	90.01%
Bao876-16-2	4695678	0.69	97.82%	40.46%	95.89%	89.52%
GN03	4312874	0.63	97.93%	41.18%	95.80%	89.30%
Xixuandahongpao-1	4245146	0.62	97.18%	41.96%	96.20%	90.13%
Bei4 NL8	4838200	0.71	98.21%	40.57%	96.09%	89.89%
Longxiaodou2hao	4750870	0.69	97.74%	40.27%	96.18%	90.09%
GN04	4662370	0.68	97.63%	41.01%	96.25%	90.23%
Bei10	4507824	0.66	98.08%	41.46%	96.11%	89.92%
Bei12 B-1	3987876	0.58	98.06%	42.16%	96.30%	90.28%
Bei23	5340322	0.78	97.95%	40.97%	96.03%	89.79%

Bei25 F174	4276244	0.62	97.67%	40.73%	96.14%	90.01%
BaoM908-15	5355770	0.78	97.85%	42.30%	96.02%	89.76%
LiaoV2baihong1hao	5001312	0.73	97.86%	41.26%	96.10%	89.89%
Jilin362	5056818	0.74	98.23%	41.04%	95.80%	89.18%
Jilv5hao	4982722	0.73	98.06%	41.43%	95.60%	88.92%
XD11	4905684	0.72	96.56%	44.79%	95.98%	89.71%
Jihong8hao	5139788	0.75	97.92%	40.47%	96.11%	89.94%
LZX022	5021368	0.73	97.48%	41.08%	96.11%	89.95%
LZX051	5604478	0.82	98.02%	41.51%	95.96%	89.65%
H90	5211446	0.76	97.61%	41.20%	96.02%	89.76%
Baoqinghong	5027728	0.73	97.81%	41.66%	95.91%	89.56%
Dacha1	4885356	0.71	97.77%	41.76%	95.92%	89.59%
FuxihongxiaodouHW 2	4931790	0.72	97.63%	40.53%	95.46%	88.68%
Kenjianhong1hao	4556758	0.67	98.15%	40.65%	95.34%	88.40%
Kenjianhong1hao-2	4875240	0.71	97.58%	42.46%	96.12%	89.98%
LJ04	5100658	0.74	98.08%	41.22%	95.93%	89.59%
Mianjin	4577898	0.67	96.84%	41.41%	96.13%	89.96%
Nonganhong	5543004	0.81	97.88%	41.01%	96.14%	89.98%
GN05	4660818	0.68	97.70%	41.36%	96.12%	89.98%
Shen10 4-C-1	5134044	0.75	97.43%	41.07%	96.14%	90.00%
Shen25 4-B-3	4607776	0.67	97.48%	40.45%	96.16%	90.01%
Titianyuanshenghong xiaodou	4992340	0.73	98.41%	40.36%	95.92%	89.52%
Zaluan2	5099600	0.74	96.80%	41.94%	96.12%	89.98%
GN06	5031190	0.73	96.90%	42.86%	96.06%	89.83%
Jihong352	4971510	0.73	96.82%	41.57%	96.41%	90.49%
Baoqinghong-2	5253372	0.77	97.21%	40.62%	96.20%	90.11%
Baihong9hao	5396394	0.79	97.22%	40.97%	95.99%	89.71%
Jihong0007-5	5268944	0.77	97.47%	40.95%	96.18%	90.08%
Jihong12	5235438	0.76	97.15%	41.22%	96.31%	90.32%
Xiaofeng2hao	5493938	0.8	97.22%	41.21%	95.51%	88.79%
Zhenzhuhong	3910262	0.57	97.35%	41.32%	95.89%	89.50%
Baihong4hao	4597818	0.67	96.95%	42.04%	95.83%	89.38%
Jihong9218	4320566	0.63	97.59%	40.94%	95.92%	89.46%
Baihong8hao	4179232	0.61	97.43%	41.47%	95.92%	89.58%
Baihong3hao	4055326	0.59	97.07%	40.55%	95.73%	89.21%
Tianjinhong	4144570	0.61	97.52%	41.54%	96.11%	89.90%
Jihong0001	4341996	0.63	97.43%	41.16%	95.99%	89.69%
Liaohong7hao	4446820	0.65	97.77%	40.95%	96.18%	90.07%
Zhenzhenhong	4337978	0.63	97.73%	41.69%	96.27%	90.26%
Liaohong7	4057822	0.59	97.78%	42.10%	96.03%	89.77%
Liaohong1hao	4294480	0.63	97.65%	41.97%	96.08%	89.85%
Liaohong3hao	4446778	0.65	97.78%	40.44%	96.13%	89.91%

Pinhong2011-18	4825498	0.7	97.83%	40.99%	96.07%	89.87%
Pinhong2011-20	4131770	0.6	97.67%	40.71%	95.70%	89.12%
Jihong0015	4898646	0.72	97.63%	41.84%	96.09%	89.83%
Jihong0001-2	4649552	0.68	97.77%	39.75%	96.29%	90.29%
Long11-203	5032562	0.73	98.02%	39.83%	96.01%	89.75%
Long11-726	4568674	0.67	97.36%	41.56%	96.10%	89.91%
1103	4148014	0.61	97.18%	44.76%	96.18%	90.05%
Baihong8hao-2	4738516	0.69	97.73%	42.00%	96.23%	90.16%
Tongza-6	3287076	0.48	97.39%	40.90%	96.60%	90.89%
Liaohong08712	4007798	0.59	97.79%	40.99%	96.20%	90.11%
Baohong200831-2	3851142	0.56	97.72%	43.21%	96.32%	90.36%
Tanghong2010-23	4106926	0.6	97.47%	43.03%	96.12%	89.92%
Tanghong2010-12	4758864	0.69	97.91%	41.13%	95.95%	89.61%
012-27	4426314	0.65	97.42%	41.83%	96.14%	90.03%
JHPX01	3976312	0.58	97.25%	42.48%	95.99%	89.68%
JHPX02	4335028	0.63	97.46%	44.21%	96.06%	89.86%
Yuhong2hao	4270762	0.62	96.87%	44.32%	96.29%	90.28%
Yuhong3hao	3849716	0.56	97.74%	39.99%	96.06%	89.86%
Jihong9218-2	4254288	0.62	97.41%	41.91%	96.15%	90.03%
Zhuangyuanhong	4420574	0.65	97.97%	41.60%	96.19%	90.11%
Jiahong1hao	5238504	0.76	97.11%	42.42%	96.08%	89.87%
Jingnong6hao	5258318	0.77	98.03%	40.84%	96.01%	89.75%
Tianjinhong-2	4458128	0.65	98.06%	40.32%	96.39%	90.46%
Baihong2hao	5770828	0.84	98.06%	40.90%	96.13%	89.96%
Jihong352-2	3730836	0.54	97.50%	42.88%	96.13%	89.94%
Xiaofeng2hao-2	3884920	0.57	97.84%	41.61%	96.26%	90.21%
012-8	5172950	0.76	97.67%	42.52%	95.93%	89.57%
012-25	3800884	0.55	97.94%	41.10%	96.33%	90.33%
Chixiaodou	4426726	0.65	98.02%	43.68%	95.84%	89.42%
Longxiaodou4hao	4261698	0.62	97.51%	43.35%	96.10%	89.95%
IIE100089	4547494	0.66	97.68%	39.37%	96.31%	90.30%
IIE100094	4466014	0.65	97.43%	43.23%	95.61%	88.91%
IIE100054	4822974	0.7	98.02%	43.43%	95.61%	88.97%
IIE100040	4920292	0.72	97.81%	39.84%	95.36%	88.52%
IIE100077	5572692	0.81	98.17%	39.69%	95.83%	89.39%
IIE100010	5017880	0.73	97.98%	39.88%	95.75%	89.19%
IIE100058	4850940	0.71	98.34%	39.54%	95.54%	88.74%
IIE100072	5402280	0.79	97.99%	40.08%	95.58%	88.92%
IIE100083	4935962	0.72	98.03%	39.83%	95.55%	88.82%
IIE100075	5250370	0.77	98.12%	39.91%	95.89%	89.54%
IIE100001	5382280	0.79	96.17%	39.31%	94.93%	87.56%
IIE100103	4043022	0.59	96.71%	39.76%	94.33%	86.59%
IIE100053	4837998	0.71	98.41%	39.31%	96.11%	89.95%
IIE100091	4229708	0.62	98.15%	39.32%	96.33%	90.35%

IIE100034	5031362	0.73	98.35%	39.58%	96.05%	89.85%
IIE100061	5810808	0.85	97.90%	40.84%	95.53%	88.68%
IIE100074	4601234	0.67	96.59%	40.04%	95.20%	88.17%
IIE100063	5748378	0.84	98.22%	39.42%	95.70%	89.01%
IIE100095	5892094	0.86	98.08%	39.35%	95.43%	88.54%
IIE100043	5676468	0.83	97.95%	39.51%	95.41%	88.49%
IIE100098	5334660	0.78	97.89%	40.13%	95.26%	88.19%
IIE100092	5331614	0.78	98.10%	39.71%	95.37%	88.40%
IIE100174	5588370	0.82	98.17%	39.44%	95.52%	88.63%
IIE100208	5454502	0.8	98.12%	40.67%	95.42%	88.52%
IIE100008	5388952	0.79	97.81%	40.83%	95.18%	88.08%
IIE100154	5545122	0.81	97.64%	39.15%	95.52%	88.60%
IIE100246	4911170	0.72	97.69%	40.60%	95.02%	87.79%
IIE100129	4832782	0.71	98.25%	39.09%	96.29%	90.28%
IIE100003	5927644	0.87	97.59%	39.74%	95.18%	88.06%
IIE100006	4784224	0.7	97.55%	39.73%	95.27%	88.25%
IIE100009	5427670	0.79	97.55%	40.37%	95.28%	88.28%
IIE100018	4226122	0.62	96.86%	39.26%	94.40%	86.66%
IIE100021	4199202	0.61	97.39%	39.53%	94.78%	87.26%
IIE100023	4510946	0.66	97.02%	39.62%	94.46%	86.75%
IIE100024	5232188	0.76	97.41%	40.21%	94.94%	87.64%
IIE100029	4659504	0.68	97.09%	39.35%	94.45%	86.74%
IIE100032	4692982	0.69	97.28%	40.09%	94.69%	87.22%
IIE100033	4719358	0.69	96.88%	39.73%	94.32%	86.50%
IIE100038	4432950	0.65	97.07%	39.70%	94.41%	86.61%
IIE100041	4977220	0.73	97.53%	39.75%	94.76%	87.25%
IIE100042	4901068	0.72	97.33%	39.76%	94.80%	87.41%
IIE100049	4553774	0.66	96.97%	39.67%	94.42%	86.68%
IIE100051	4863854	0.71	97.19%	39.81%	94.46%	86.67%
IIE100055	4432146	0.65	97.04%	39.49%	94.20%	86.28%
IIE100056	5174500	0.76	97.31%	40.06%	94.69%	87.16%
IIE100057	5058354	0.74	97.13%	39.84%	94.52%	86.86%
IIE100069	5469444	0.8	97.40%	40.15%	94.97%	87.66%
IIE100071	5031680	0.73	97.38%	39.77%	94.63%	87.05%
IIE100073	5035166	0.74	96.98%	39.76%	94.80%	87.39%
IIE100082	4758146	0.69	97.22%	39.79%	94.62%	87.04%
IIE100085	5037160	0.74	97.41%	39.75%	94.68%	87.14%
IIE100086	4910006	0.72	96.58%	39.72%	94.73%	87.25%
IIE100087	4813092	0.7	97.00%	39.58%	94.74%	87.25%
IIE100088	4779350	0.7	95.41%	39.60%	94.21%	86.30%
IIE100099	5205734	0.76	97.49%	39.90%	95.05%	87.83%
IIE100104	5117020	0.75	97.47%	39.93%	94.96%	87.68%
IIE100105	4932074	0.72	97.14%	40.20%	94.76%	87.30%
IIE100108	4675790	0.68	97.40%	39.69%	94.82%	87.42%

IIE100112	5443406	0.79	97.28%	40.77%	94.98%	87.65%
IIE100113	4232064	0.62	97.58%	39.42%	94.92%	87.59%
IIE100114	5057124	0.74	97.55%	40.21%	95.16%	88.04%
IIE100115	4920120	0.72	97.51%	39.44%	94.98%	87.69%
IIE100119	5460676	0.8	97.57%	40.14%	95.30%	88.31%
IIE100120	4384196	0.64	96.55%	39.32%	94.41%	86.70%
IIE100122	4663506	0.68	97.63%	39.60%	95.10%	87.93%
IIE100238	4678574	0.68	97.43%	40.09%	94.93%	87.62%
**Total**	855255328	124.87	_	_	_	_
**Average**	4804805.213	0.70	97.59%	40.92%	95.67%	89.07%,

Table S2 Rust resistance evaluation of adzuki bean accessions at seedling and adult stages

Samples	Seedling Stage (Infection Type)	Adult Stage (Disease Index)
TL01	2	51.61
Jilin373	4	62.44
Bao876-16	4	17.68
Hongdou XD04-07	4	94.42
TL02	3	13.99
TL03	4	100.00
Jian265	4	9.19
LZX066	4	72.90
LiaoV5	3	17.59
QH6	4	100.00
Jihanhongxiaodou	2	40.66
Bei6 JN003	2	25.56
Longkenzaohong	2	14.60
TL05	2	100.00
TL06	1	32.45
Jihong6hao	3	35.49
Bei13 F015	4	100.00
QH1	4	46.72
QH3	2	28.43
QH4	4	100.00
QH7	3	37.06
LJ01	2	21.09
QH5	3	32.22
QH2	3	25.25
LJ02	3	36.04
HongdouXD04-02	2	24.09

LJ03	2	25.41
GN01	4	27.46
GN02	3	13.08
HongdouXD04-09	2	6.11
Pinhong23129-1	3	5.05
Bao876-16-2	3	53.64
GN03	4	14.70
Xixuandahongpao-1	2	6.06
Bei4 NL8	2	14.93
Longxiaodou2hao	4	36.16
GN04	3	61.28
Bei10	3	33.96
Bei12 B-1	3	73.83
Bei23	3	61.45
Bei25 F174	3	22.12
BaoM908-15	3	100.00
LiaoV2baihong1hao	3	100.00
Jilin362	2	29.69
Jilv5hao	3	75.64
XD11	3	100.00
Jihong8hao	3	50.00
LZX022	3	41.72
LZX051	2	58.59
H90	4	40.09
Baoqinghong	3	100.00
Dacha1	3	30.53
FuxihongxiaodouHW2	4	6.31
Kenjianhong1hao	3	100.00
Kenjianhong1hao-2	4	59.68
LJ04	2	34.85
Mianjin	3	10.82
Nonganhong	4	18.48
GN05	3	100.00
Shen10 4-C-1	3	36.39
Shen25 4-B-3	3	16.58
Titianyuanshenghongxiaodou	2	77.17
Zaluan2	3	71.82
GN06	2	78.38
Jihong352	2	24.37
Baoqinghong-2	3	92.82
Baihong9hao	3	100.00
Jihong0007-5	2	20.14
Jihong12	1	13.69
Xiaofeng2hao	2	100.00

Zhenzhuhong	4	10.91
Baihong4hao	3	15.91
Jihong9218	2	10.10
Baihong8hao	4	53.00
Baihong3hao	3	100.00
Tianjinhong	4	33.13
Jihong0001	2	14.77
Liaohong7hao	2	4.80
Hongzhenzhu	3	6.46
Liaohong7	2	37.11
Liaohong1hao	4	6.94
Liaohong3hao	4	5.96
Pinhong2011-18	2	26.11
Pinhong2011-20	4	37.78
Jihong0015	3	12.78
Jihong0001-2	3	9.09
Long11-203	3	55.56
Long11-726	3	29.04
1103	4	100.00
Baihong8hao-2	3	NA
Tongza-6	4	50.30
Liaohong08712	4	14.44
Baohong200831-2	2	19.10
Tanghong2010-23	4	100.00
Tanghong2010-12	3	100.00
012-27	2	42.98
JHPX01	4	14.65
JHPX02	1	49.23
Yuhong2hao	3	21.90
Yuhong3hao	4	76.29
Jihong9218-2	NA	NA
Zhuangyuanhong	4	13.97
Jiahong1hao	3	68.10
Jingnong6hao	4	36.66
Tianjinhong-2	2	48.74
Baihong2hao	4	54.35
Jihong352-2	3	100.00
Xiaofeng2hao-2	3	78.03
012-8	3	47.78
012-25	4	100.00
Chixiaodou	4	100.00
Longxiaodou4hao	4	21.31
IIE100089	4	91.51
IIE100094	NA	90.24

IIE100054	3	88.94
IIE100040	3	75.94
IIE100077	2	87.64
IIE100010	4	91.76
IIE100058	3	3.18
IIE100072	4	71.05
IIE100083	2	58.70
IIE100075	3	44.47
IIE100001	4	58.30
IIE100103	1	0.20
IIE100053	3	47.97
IIE100091	4	95.19
IIE100034	3	84.64
IIE100061	4	63.39
IIE100074	0	1.87
IIE100063	2	96.57
IIE100095	3	79.24
IIE100043	2	46.38
IIE100098	4	49.17
IIE100092	3	82.78
IIE100174	2	84.06
IIE100208	3	79.51
IIE100008	NA	94.85
IIE100154	2	90.27
IIE100246	NA	88.55
IIE100129	2	92.97
IIE100003	3	92.44
IIE100006	3	34.44
IIE100009	3	32.73
IIE100018	3	70.81
IIE100021	2	56.31
IIE100023	3	43.69
IIE100024	3	38.18
IIE100029	2	83.74
IIE100032	2	52.37
IIE100033	1	27.22
IIE100038	2	20.39
IIE100041	3	0.00
IIE100042	3	39.20
IIE100049	1	65.66
IIE100051	1	30.93
IIE100055	1	78.16
IIE100056	2	70.76
IIE100057	3	60.29

IIE100069	3	20.71
IIE100071	2	73.81
IIE100073	NA	84.66
IIE100082	1	15.72
IIE100085	3	36.01
IIE100086	3	51.35
IIE100087	2	56.50
IIE100088	2	66.93
IIE100099	1	0.00
IIE100104	2	38.57
IIE100105	3	37.75
IIE100108	2	61.98
IIE100112	2	8.78
IIE100113	3	78.96
IIE100114	3	86.84
IIE100115	3	86.41
IIE100119	NA	31.12
IIE100120	3	60.19
IIE100122	2	35.67
IIE100238	3	39.14

Note: NA indicates that data were not available for the corresponding accession.
